# Congenital intestinal stenosis and Hirschsprung’s disease: two extremely rare pathologies in a newborn puppy

**DOI:** 10.1186/s12917-019-1806-z

**Published:** 2019-03-13

**Authors:** Angélica Morales-Miranda

**Affiliations:** Department of Reproductive Biology, National Institute of Medical Sciences and Nutrition Salvador Zubirán, Avenue. Vasco de Quiroga 15 Col. Belisario Domínguez, Section XVI, Tlalpan, 14080 México City, Mexico

**Keywords:** Congenital intestinal stenosis, Enteric nervous system, Ganglion cells, Hirschsprung’s disease

## Abstract

**Background:**

Hirschsprung’s disease (HSCR) is a common congenital malformation of the enteric nervous system (ENS). During fetal development, ganglion cells of the ENS are derived from neural crest cells that migrate to the bowel. These cells reside principally in two ganglionated plexus: 1) The *myenteric plexus*, extending from the esophagus to the anus, and 2) *submucous plexus*, extending from the duodenum to the anus. In large animal species, there is a third plexus called Henle’s or Schabadasch’s plexus.

ENS ganglion cells play a key role in normal gastrointestinal motility, respond to sensory stimuli and regulate blood flow. Both plexus show a high degree of independence from the central nervous system. Alterations in the embryonic development of the ENS can induce multiple pathologies in animal models and humans.

**Case presentation:**

The present case was a female the fifth born in a litter of 5 puppies. At about 2–3 weeks of age, she suffered from abdominal distension, pain, and constipation. At approximately 8–10 weeks of age, the puppy started to vomit abundantly, and the regurgitated food appeared undigested. Progressive abdominal distention was observed, with quite visible peristaltic movements and more frequent vomiting episodes. The abdominal radiographs, based on AP and side projections, revealed an enlargement of the abdominal diameter and an increased width in the epigastric region. At 12 weeks of age, exploratory surgery revealed a stenotic segment in the jejunum, followed by a small transition zone and then a significantly reduced diameter. Immunohistochemical examinations were performed using antibodies against calretinin, S-100 protein, CD56, neuron specific enolase (NSE) and synaptophysin, which are the biological markers for diagnosing HSCR.

**Conclusion:**

A reduced number of ganglion cells (1–3 cells per ganglion) were found. There was no specific staining pattern for many of these; while for others, the pattern was compatible with HSCR. Surgical intervention to remove the stenotic section prolonged the life of the puppy for 13 years. Extremely rare pathologies such as that discussed herein should be studied to understand the pathophysiology and be able to diagnose small species in veterinary medicine in a timely fashion. To our knowledge, this is the first report of congenital intestinal stenosis and Hirschprung’s disease in a newborn puppy.

**Electronic supplementary material:**

The online version of this article (10.1186/s12917-019-1806-z) contains supplementary material, which is available to authorized users.

## Background

The embryonic development of the enteric nervous system (ENS) is a complex process that begins with the migration of neural crest cells, followed by their proliferation and differentiation in the wall of the gastrointestinal tract. There are key points during this process in which cells must undergo certain transformations for the attainment of a functional ENS.

The coordinated action of neural crest cells is critical for the development and functioning of the intestinal microenvironment [1, 2]. Some studies have demonstrated that a specific number of cells is required for the migration through the intestinal mesenchyme to begin. This migration occurs in the craniocaudal direction in response to diffusible molecules in cell-cell interactions.

The expression of transcription factors induces cell arrest, proliferation, and differentiation for neuronal and/or glial phenotypes [3–5]. During this process, the vital function of motility develops in the gastro-intestinal tract. Gut motility is a complex process mediated by the interaction between intestinal smooth muscle, interstitial cells of Cajal, and a diverse range of neuronal phenotypes (characterized by neurotransmitters) in the ENS. All these cells are essential for generating the two main types of contractions in the gut: segmentation and peristaltic waves. These two mechanisms occur in the absence of extrinsic innervation and require intact submucous (Meissner) and myenteric (Auerbach) nerve plexus along the gastrointestinal tract [6, 7]. In large animal species, there is a third plexus called Henle’s or Schabadasch’s plexus, which is adjacent to the luminal side of the circular muscle coat. According to the species, these plexuses differ in number, shape and size of ganglia, number and size of neurons and number and diameter of nerve fibers [8–12].

Hirschsprung’s disease (HSCR) is a congenital disorder involving the absence of ganglion cells in the submucous and myenteric nerve plexus, which leads to an obstruction of the gastrointestinal tract. The first presentation of HSCR symptoms is attributed to a Danish pediatrician, Harald Hirschsprung, MD., (1830–1916) [13]. Nevertheless, similar symptoms were reported by Italian physician Domenico Battini, who did the follow-up on a case of severe constipation ten years previously [14].

Today, more than a century after the death of Harald Hirschsprung, significant progress has been made in characterizing the molecular processes involved in the pathogenesis of HSCR [15, 16]. The disease is located in the last portion of the gastrointestinal tract (the rectum and sigmoid colon) in 80% of cases, while extending to other colon segments in 20% of cases [17–19]. HSCR is extremely rare in the small intestine, as a manifestation of total enteric aganglionosis is almost impossible. The report on “skip segment” Hirschsprung’s disease (SSHD) describes a zone of aganglionosis within the normally ganglionated intestine. Other variants of intestinal sections from patients with HSCR-like symptoms have sometimes evidenced hypoganglionosis (a decrease in the number of ganglion cells in both plexus) or “immature” neuronal cells. The pathogenesis of these presentations cannot be explained embryologically [20–22].

Multiple reports have associated HSCR with malformations that occur in polygenic disorders [23, 24], such as Down syndrome [25], Waardenburg syndrome [26], Bardet-Biedl syndrome, gastrointestinal malformations (Meckel’s diverticulum, colonic atresia and anorectal defects), and congenital heart disease (septal defects or ductus arteriosus) [27].

Genetic studies have suggested that autosomal dominant Mendelian inheritance with a mutation in RET on chromosome 10 (locus at long arm 10q11.2q21.2), now referred to as ***HSCR1***, has a 250-kb DNA interval containing the RET proto-oncogene. This mutation is responsible for approximately 40% of the cases of Hirschsprung’s disease [28].

RET is a cell-surface protein possessing an extracellular ligand-binding domain, a single hydrophobic transmembrane region, and an intracytoplasmic tyrosine kinase domain. It plays a crucial role during the development of the excretory system and the ENS [29, 30]. Recently a second ***HSCR2*** locus was identified on chromosome 13 (the deletion of 13q), which is linked to congenital anomalies [31]. Although mutations of the RET tyrosine kinase gene are still the only known cause of the disorder, there are reports of at least 12 genes that determine the final phenotypic expression of the pathogenesis. These genes include the endothelin converting enzyme ECE-1*(1p36.1)* [32], the glial-cell-line-derived neurotrophic factor GDNF *(5q12–13.1)* [33, 34], endothelin receptors type B or EDNRB *(13q22)* [35], NTN *(19p13.3)* [36], endothelin 3 EDN3 *(20q13.2–13.3)* [37], and the gene encoding the Sry-related transcription factor SOX10 *(22q13.1)* [38]. Analysis of the afore mentioned gene products in experimental animal models and cell cultures has led to increasing clarity about the signaling pathways involved during specific embryonic stages that direct the spatial arrangements and differentiation of enteric cells derived from the neural crest [39–43].

Recent studies show growth factors as key to the development of Hirschsprung’s disease, these being fundamental for the survival, differentiation, and function of enteric neurons and glia during fetal gut development [44, 45]. Among such growth factors are brain-derived neurotrophic factor (BDNF), nerve growth factor (NGF) and neurotrophin-3 (NT-3) [46–48].

Growth factors are ligands for the tyrosine kinase family of receptors (RTKs), encoded by Trk proto-oncogenes. These ligands and receptors are both essential components for interactions between neurotrophins and neuronal cells.

Little is known about the relation of Hirschsprung’s disease to other congenital intestinal malformations, such as stenosis or atresia. Congenital malformations encompass a wide variety of alterations during fetal development. In 1955, Louw et al. reported the possibility that an occlusion in the blood supply or an episode of ischemia triggers alterations in gut developmental [49, 50]. Experiments using animal models have demonstrated that a permanent occlusion in the blood supply can be one of the causes of atresia, stenosis and similar pathologies [51].

We herein present a clinical case of a newborn pup (Yorkshire Terrier) which was first taken to the veterinarian due to vomiting, enormous distension of the abdomen and constipation. The initial diagnosis was difficult, since this is an extraordinarily rare disease in veterinary medicine. However, the clinical data was similar to that found with HSCR in humans or congenital enteric neuropathies.

## Case presentation

The dog was the female the fifth born in a litter of 5 puppies. Compared to her siblings, she exhibited the smallest size and lowest weight from birth. At about 2–3 weeks of age, she suffered from abdominal distension, pain, and constipation. The veterinarian recommended rectal stimulation and cisapride (prepusid) 0.5 ml every 8 hours for 2 days. With great effort, the puppy defecated once a day after stimulation, and her stools had normal characteristics. At approximately 8–10 weeks of age, she began a diet of semisolid food consisting of small portions eaten 3–5 times a day, which caused her to start vomiting abundantly.

The regurgitated food appeared undigested. Blood tests, stool analysis, and liver function tests were normal, giving no clues for a diagnosis. Anti-parasitic treatment was initiated but did not improve her symptomatology. Multiple diets were implemented, including soymilk and pancreatic enzymes prescribed for supposed lactose intolerance or pancreatic insufficiency.

**Fig. 1:**
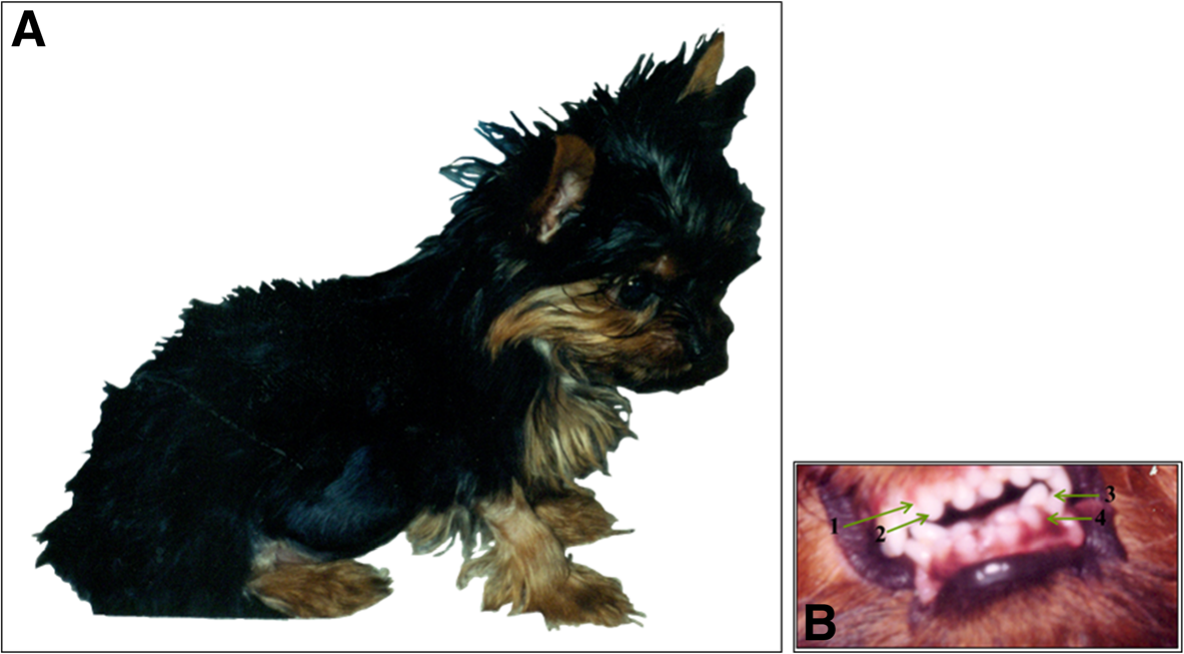
Photograph of the puppy under study at approximately 10 weeks of age, with an enormous abdominal distension (**a**) and a second row of teeth (**b**)

Progressive abdominal distention was observed (Fig. 1a), with quite visible peristaltic movements and more frequent vomiting episodes. During the clinical examination, the second row of upper and lower teeth were noted (Fig. 1b). Different veterinarians were consulted, and one suggested a probable alteration in intestinal motility. Regulators of peristalsis (metoclopramide, dimeticone, and lactulose) were prescribed. On some occasions, third generation quinolones were administered to treat bacteremia. None of the treatments or schemes led to an improvement in the dog, resulting in a severe deterioration of her general condition accompanied by fever.

**Fig. 2:**
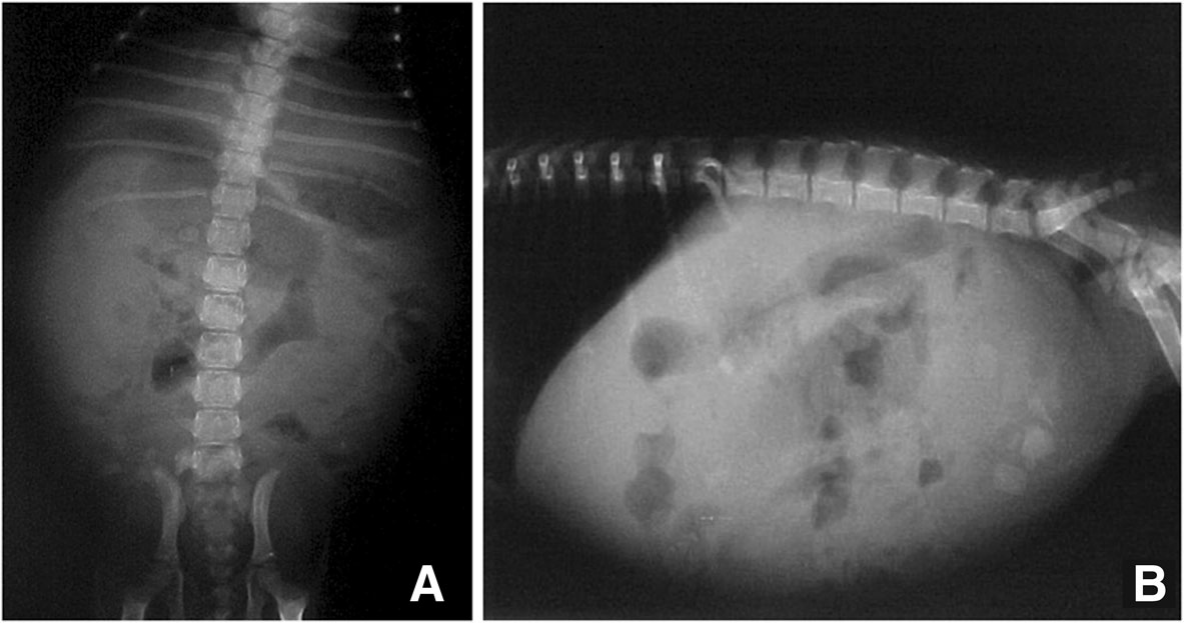
Radiological features of abdominal distension, showing an increase in abdominal diameter and the presence of gas in the intestinal loops, as can be appreciated in the anteroposterior view (**a**) and lateral view (**b**)

**Fig. 3:**
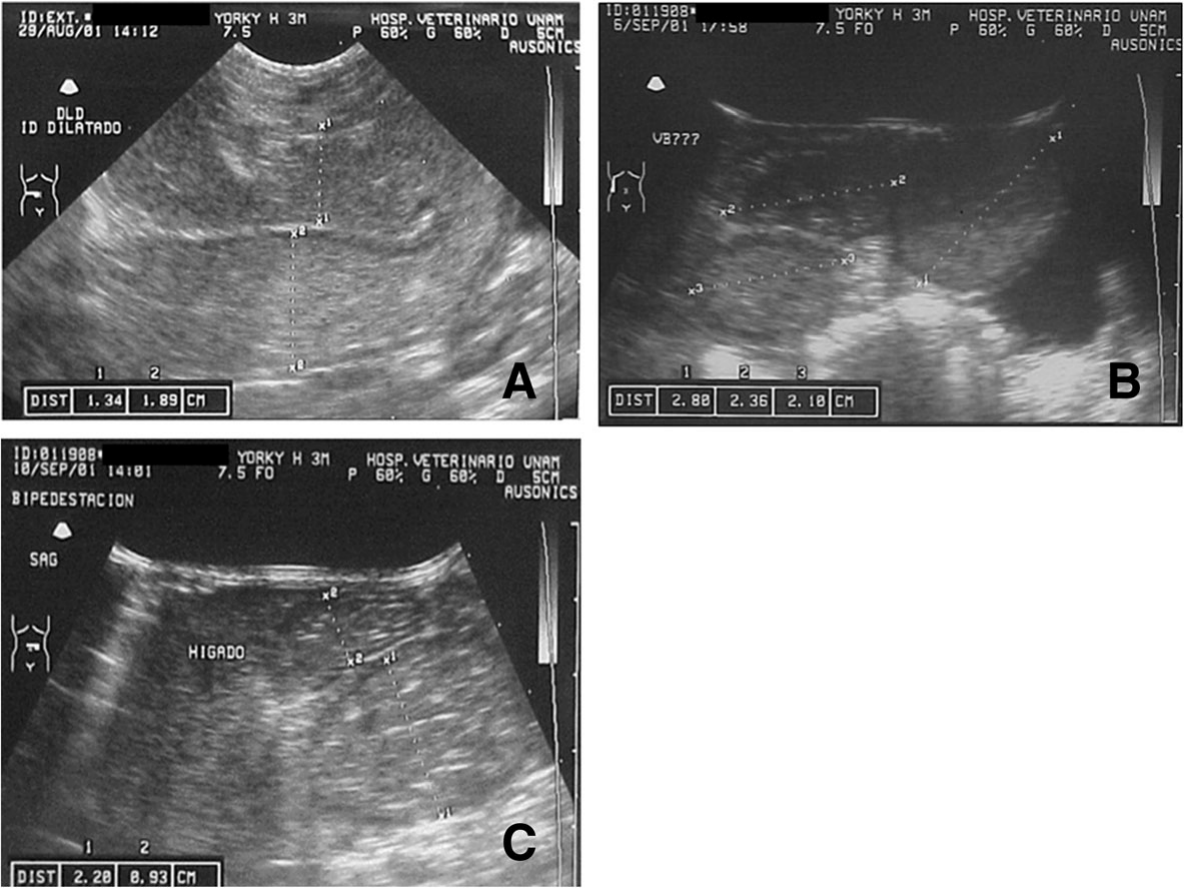
The abdominal ultrasound reveals an increase in the diameter of the small intestine, from 1.34 to 2.20 cm. There is enhanced echogenicity, the presence of liquid and gas, and a thin wall in the loops of the intestine (**a-b**). The liver and gallbladder are within normal limits (**c**)


The abdominal radiographs, based on AP and side projections, revealed an enlargement of the abdominal diameter with the presence of gas in the intestinal wings, and an increased diameter in the epigastric region (Fig. 2a and b). Abdominal ultrasonography on distinct dates evidenced dilated bowel loops with a great amount of echogenic content, a thin wall in the intestinal wings, and free liquid in the intra-abdominal cavity (Fig. 3a, b and c and Additonal file 1). The liver, biliary ducts, and kidneys had normal characteristics.

**Additional file 1:** Abdominal ultrasound performed before surgery. An increase in small intestine movements are observed. (WMV 1950 kb)


**Fig. 4:**
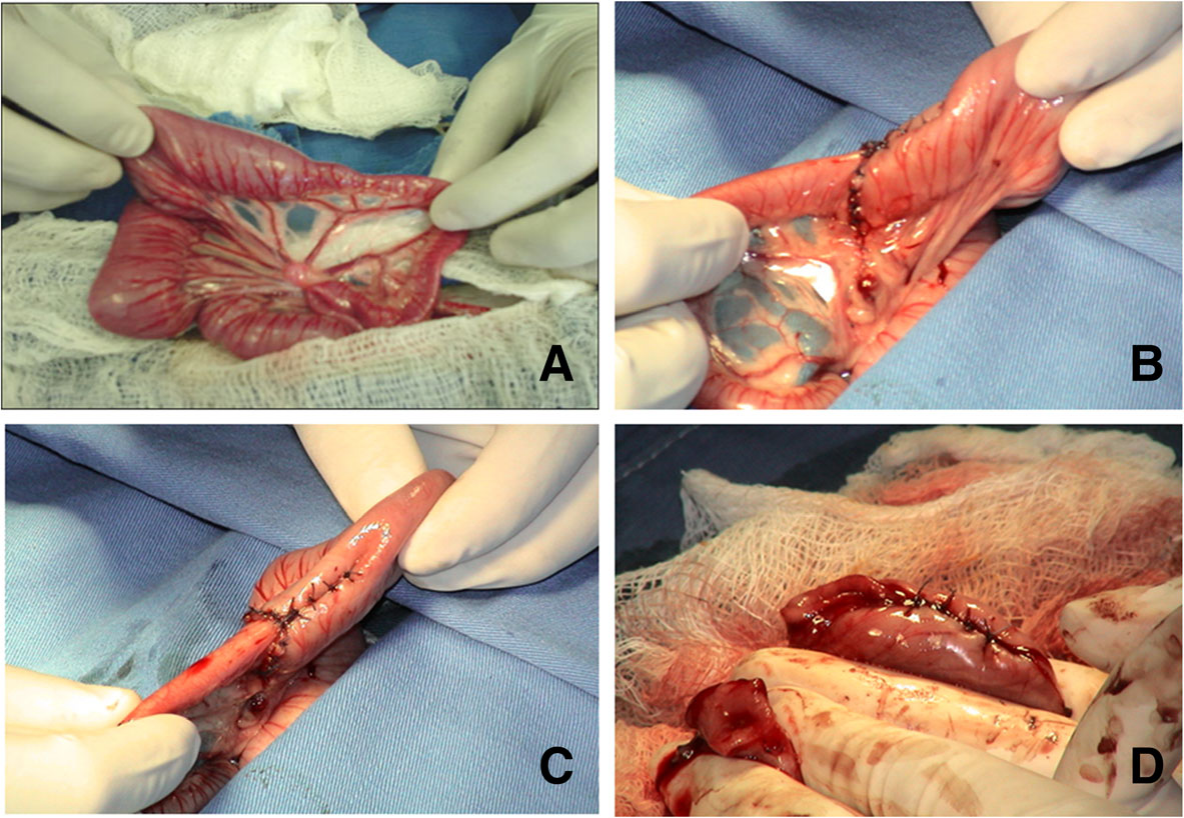
Photographs taken during surgery illustrate a segment of approximately 12 cm of the intestine, corresponding macroscopically to the jejunum. A reduction in diameter can be observed before the segment of stenosis (**a**). The subsequent images demonstrate the elimination of this segment, and then the technique used for the reduction of the hypertrophied segment and the joining of the two ends of the intestine (**b-d**). The proximal diameter had a circumference of 0.5 cm, while the distal side measured 3–4 cm

After multiple studies and many visits to veterinarians, there was a recommendation of exploratory surgery, which was performed when the puppy was about 12 weeks old. During the examination of the abdomen, a stenotic segment of approximately 12 cm was identified in the jejunum, followed by a small transition zone and then a significantly reduced diameter (Fig. 4a). Resection of this segment and surgical management of a jejunoileonstomy was carried out (Fig. 4b and c).

**Fig. 5:**
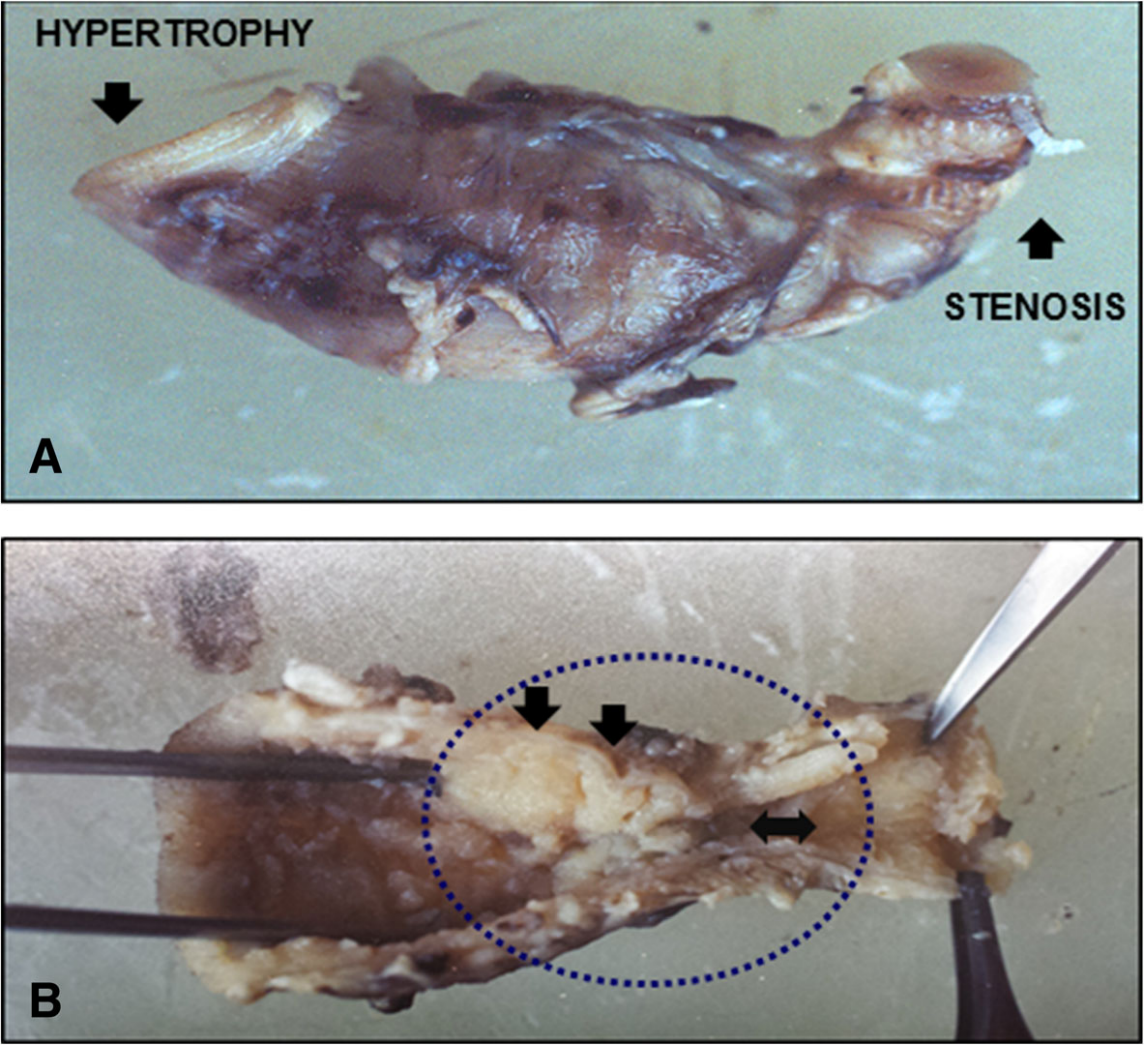
A fragment of the small intestine obtained during surgery (**a**). Upon opening this segment, it was found that fibrotic tissue occupied a considerable part of the intestinal lumen (without completely occluding it) and was present in the transition zone (**b**)

The circumference of the stenotic segment was approximately 5 mm and of the dilated proximal extreme about 3 cm (Fig. 4d, and Fig. 5a). The open segment shows fibrotic tissue that occludes this small transition zone (Fig. 5b). No other abdominal injury was present. The abdominal fascia was closed with 3–0 polyglactin and the skin with 3–0 nylon suture.

**Fig. 6:**
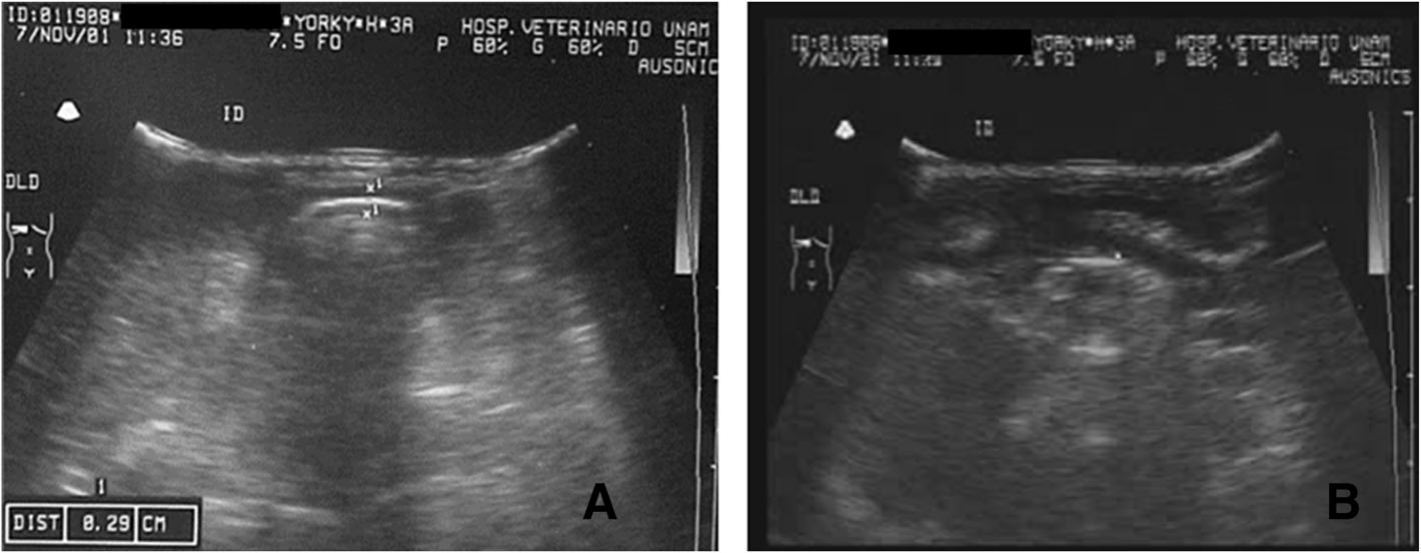
The ultrasound image of the real size of the intestine reveals a greatly reduced diameter (0.29 cm) after surgery (**a**) compared to before surgery. In the video, normal intestinal movements can be appreciated (**b**)


The postoperative period was uneventful, and the puppy was discharged on day four post-surgery without complications. She was given a soft diet, and her weight subsequently increased. Her feces content took on a healthy form. A post-surgical ultrasound revealed bowel loops with a diminished diameter and normal peristaltic movements (Fig. 6 and Additional file 2). The puppy lived the next 13 years without digestive problems.

**Additional file 2:** Abdominal ultrasound performed after surgery. Restoration of normal peristalsis. (WMV 1950 kb)


We hypothesized that abdominal distention, vomiting, and constipation in the early stages of the life of the dog pup could evidence a disorder similar to human disease. Thus, the symptomatology of the disorder was compared to pediatric diseases of the digestive tract. The specific combination of clinical signs and symptoms, in this case, led to a tentative diagnosis of Hirschsprung’s disease. However, the discovery of jejunum stenosis made the diagnosis of HSCR less likely. Stenosis is an extremely rare congenital condition that is usually located in the small intestine, as presently found. It is generally resolved with a relatively safe surgery. Accordingly, a primary anastomosis was herein performed with a maximal diameter variance of 3:1 (proximal: distal) [52]. A small surgically resected fragment was obtained and embedded in a single block of paraffin, which was sliced and placed on slides (Fig. 5). H&E staining was used, since it is the diagnostic method of choice for identifying ganglion cells in both plexus [53].

Whereas histopathological analysis revealed mucosa without alterations, the submucosa expanded in varying degrees through fibrous connective tissue, in the transverse and longitudinal muscular layer, and in the adipose tissue adjacent to the serosa.

**Fig. 7:**
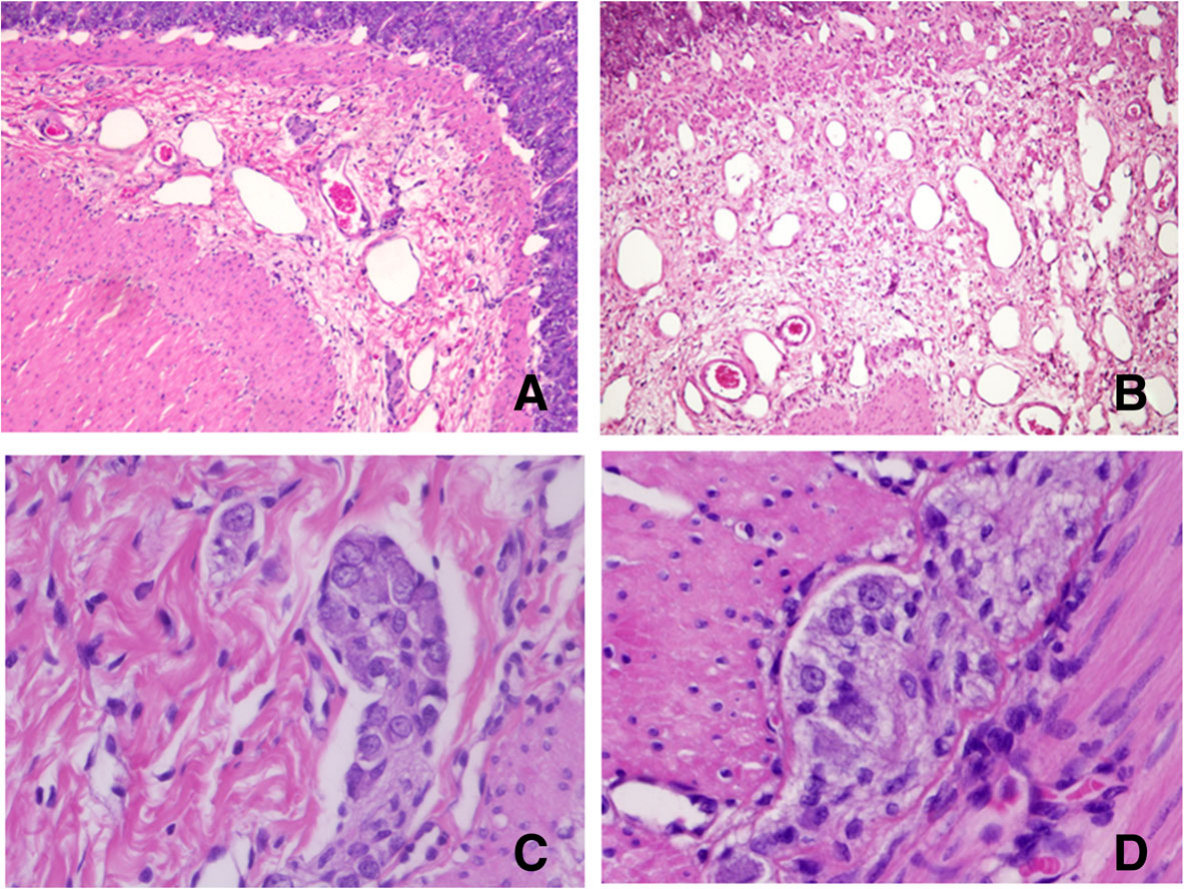
Histological aspect of small intestine stained with H&E. Panel **a** and **b** show changes in the submucous, such as fibrous connective tissue, edema, inflammation and angiogenesis. Degenerative changes of ganglion cells can be appreciated in both plexus (panels **c** and **d**) Magnification, × 10 (panels **a-b**) and × 40 (panels **c-d**)

Additionally, there was neoformation of blood vessels and edema (Fig. 7a and b). Degenerative ganglion was mild to moderate in both plexus. The ganglion cells had an oval nucleus with fine granular or euchromatin (degeneration) and eosinophilic cytoplasm (Fig. 7c and d). To confirm this observation, various pathologists were consulted. After making multiple cuts, all the specialists coincided with the finding of ganglion cells along the fragment (hypertrophy-fragment, transition zone, and stenotic fragment), and that these had the previously described morphological characteristics. The method used for immunohistochemistry (IHC) has been previously described in detail by Morales et al. [54]. Calretinin, S-100 protein and CD56 antibodies have been used for studies in different animal species including dogs [55, 56]. Cross reactivity of antibodies among different species is a standard practice for the selection of other neural immunomarkers [57–59].

**Fig. 8:**
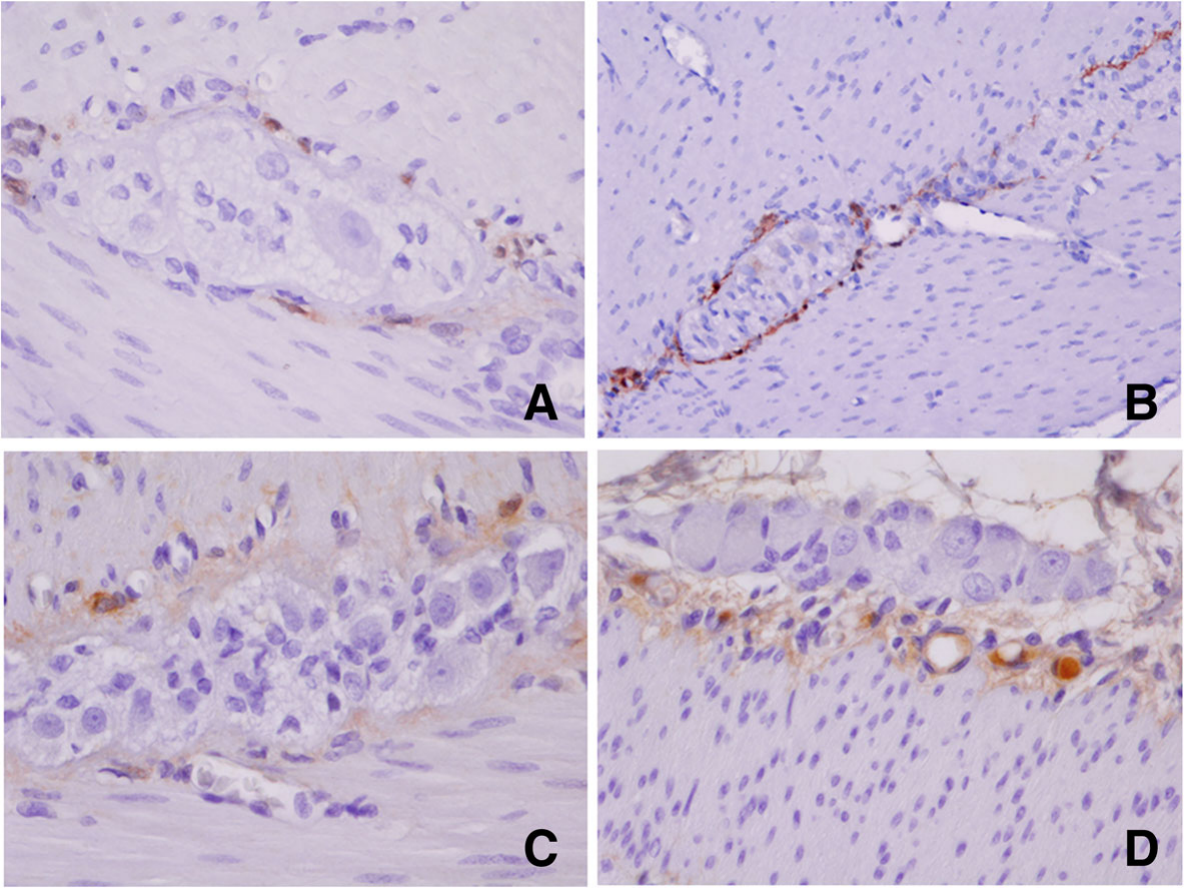
Representative microphotograph showing a total absence of calretinin staining in ganglion cells. In every panel, we can observe positivity to peripheral neurones, Schwann cells, and axons in the myenteric plexus. Magnification, × 40 (panels **a**, **c-d**) and × 10 (panel **b**)

**Fig. 9:**
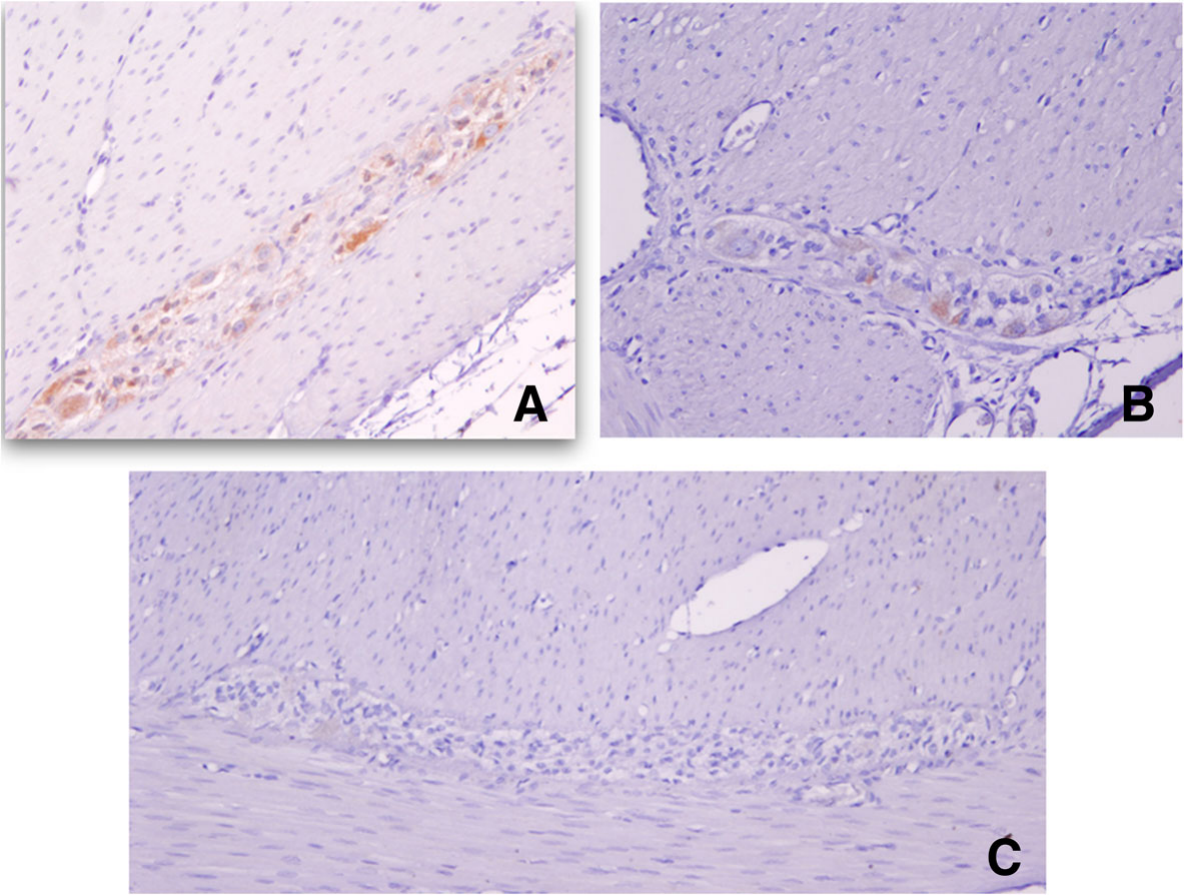
Representative light micrographs for S-100 protein. Panels **a** and **b** show a moderate staining in Schwann cells in the myenteric plexus. A total absence of ganglion cells is depicted in panel **c.** Magnification, × 20 (panels **a**, **b-c**)

**Fig. 10:**
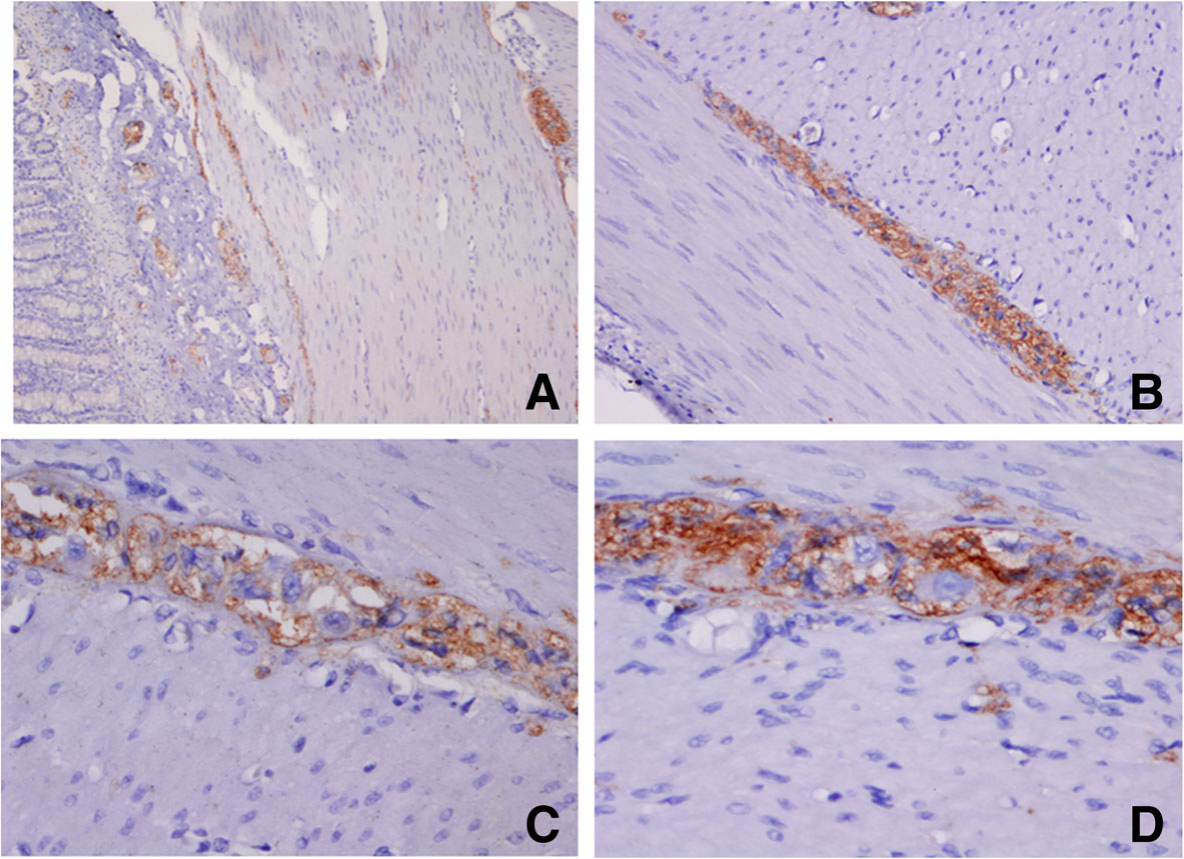
Immunohistochemical analysis of CD56. Panel (**a**) show images with positive immunostaining in both plexus. In panel (**b**), an intense staining is present in the cytoplasm of glial cells. Panels (**c-d**), show images of a reduced number of ganglion cells in the myenteric plexus. Magnification, × 10 (panel **a**), × 20 (panel **b**) and × 40 (panels **c-d**)

**Fig. 11:**
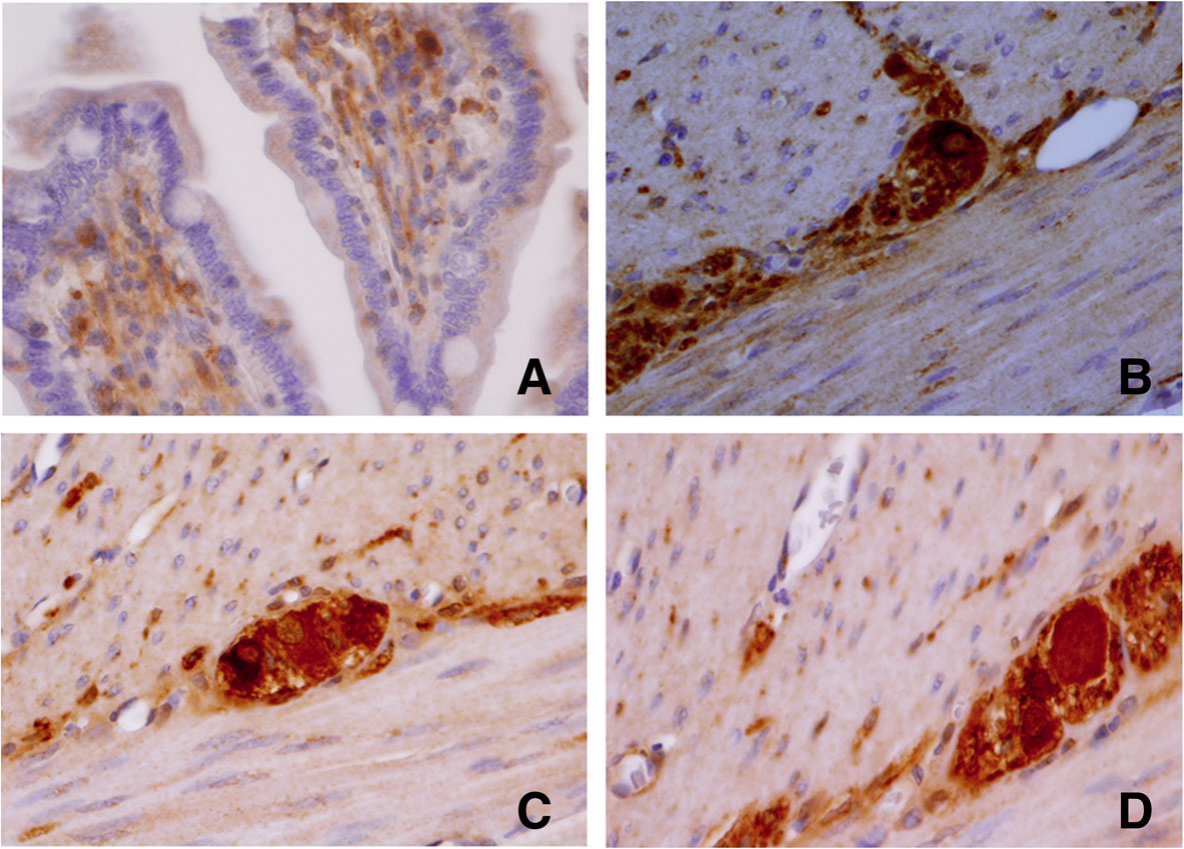
The NSE shows a strong intracytoplasmic positivity in neuroendocrine cells of the intestinal mucosa (panel **a**). In panels **b**, **c** and **d**, ganglion cells display intense cytoplasmic and nuclear staining in the myenteric plexus. Magnification, × 40 (panels **a-b** and **c-d**)

**Fig. 12:**
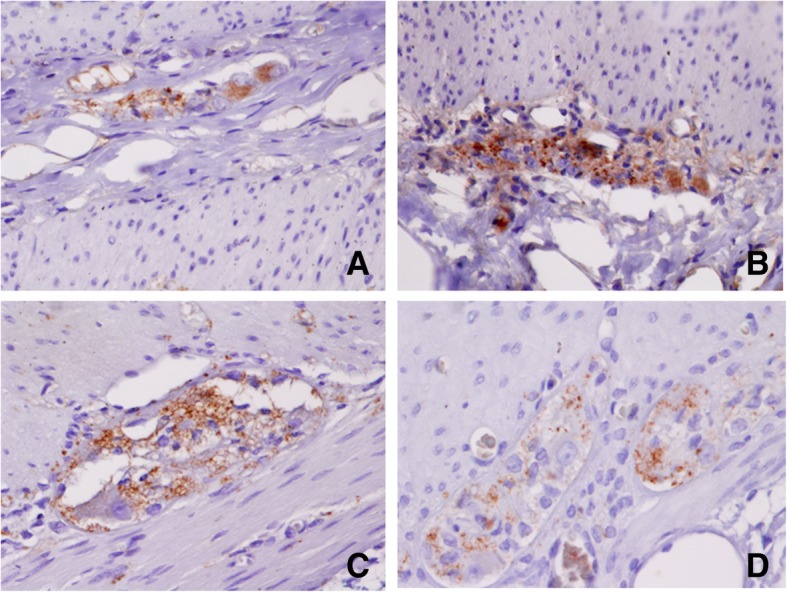
Synaptophsin (SYN) labeling. Images in panels **a-b** show moderate immunolabeling in cells located inside the ganglion. However, the ganglion cells were not immunolabeled panels as shown in panels **c-d**. Magnification, × 20 (panel **b**) and × 40 (panels **a**, **c-d**)

Briefly, a small fragment of stenosed intestine was fixed in 4% *w*/*v* formaldehyde in phosphate-buffered saline (PBS), for 24 h. Paraffin-embedded tissue sections (5-μM thickness) were mounted on glass slides (DAKO Corp., Santa Barbara, CA, USA), the sections were dewaxed in xylene (Sigma-Aldrich, Saint Louis, MO, USA) and rehydrated in graded ethanol solutions. Histopathological evaluation of some sections was stained with H&E (Sigma-Aldrich, Saint Louis, MO, USA). Slides were heated by microwave radiation for 10 min in 0.01 M citrate buffer (pH 6.0) for antigen retrieval. The activity of endogenous peroxidase was blocked with 0.3% H_2_O_2_/methanol at room temperature for 30 min. To prevent nonspecific antibody binding, sections were preincubated with protein blocking buffer diluted in PBS/bovine serum albumin (BSA; 1%; Sigma-Aldrich) for 60 min. Slides were then incubated with respective primary antibodies to anti-Calretinin (clone: EP1798, dilution 1:300), anti-CD56 (clone 123C3.D5, dilution 1:200), anti-S100 protein (clone: 4C4.9, dilution 1:200), (BioSB, Santa Barbara, CA, USA), anti-NSE (ab79757, dilution 1:200), (Abcam, Burlingame, CA, USA) and anti-Synaptophysin (clone SY38, dilution 1:100), (DAKO Corp., Santa Barbara, CA, USA), overnight at 4 °C. Subsequently, the slides were washes with PBS for 5 min each, the primary antibody was detected with the appropriate secondary antibody (Mouse/Rabbit-ImmunoDetector-HRP Cat. BSB0003-BioSB) at 1:100 dilution for 60 min at 37 °C. The sections were washed for 5 min, and incubated using 3, 3′-diaminobenzidine tetrahydrochloride (DAB) as chromogen (Zymed®/Invitrogen Inc., CA, USA). Finally, the slides were rinsed in distilled water and nuclear counterstaining with Mayer’s hematoxylin (MHS-1, Sigma-Aldrich Co., St. Louis, MO, USA). The sections were mounted and coverslipped with a synthetic mounting medium (Entellan; Merck, Darmstadt, Germany; OB046327). Photographs were taken on a Nikon-Eclipse 80i microscope coupled to a Nikon digital sight camera (Melville, NYC, USA). In each case, negative controls without the primary antibody were included (data not shown). The results of the IHC shown herein represent the general pattern of staining evidenced in the respective areas. Calretinin did not positively stain ganglion cells, observe positivity to peripheral (neurons, Schwann cells, and axons) in the myenteric plexus (Fig. 8). The reactivity of the ganglion cells to S-100 protein was negative in both plexus (Fig. 9). The staining pattern for CD56 was moderately immunopositive in glial cells throughout the submucous and in the myenteric plexus, but negative in ganglion cells (Fig. 10). Two new markers, NSE and synaptophysin, have been extensively used for diagnosis of Hirschsprung’s disease. The NSE staining was herein found to be strongly positive in the epithelium as well as the nerve cells and nerve fibers. Intense intracytoplasmic dysfunction was exhibited in the ganglion cells of the myenteric plexus. (Fig. 11). Compared to the ganglion cells, many neurons exhibited a stronger signal for NSE during embryonic development and later stages [60, 61]. For synaptophysin, on the other hand, staining was unspecific around glia cells (Fig. 12). Unfortunately, the complete stent fragment could not be obtained for this assay.

## Discussion and conclusions

Studies that explore the possible similarity of pathogenesis in distinct species are scarce. Such reports give comparisons of human diseases to those of horses, mice, calves and others domestic species like cats [62–64]. Regarding similarities in the development of the ENS for distinct species, many aspects coincide for all vertebrate classes, including mammals, birds, reptiles, amphibians, and fish [65–67]. Indeed, species-specific differences are minimal. Various mutations have been characterized in animal models and humans. It is generally accepted that genetic mutations in some animal models lead to developmental defects in neural crest cell migration, differentiation and/or survival.

A careful search of the literature was conducted in respect to the anomalies associated with double row of teeth. There are few descriptions of such a condition in the literature that demonstrate a similarity between species [68, 69]. Even though genetic tests for identifying mutated chromosomes are commercially available for humans and horses, this is not the case for other species. Unfortunately, no other information is available because genetic studies in veterinary medicine are sporadic.

The present case report has some limitations that significantly reduce the certainty of a parallel between this extremely rare disease in humans and animals. Although the dog pup was examined from the time of birth, little control was exercised over some clinical variables, and the surgical intervention was not based on a thorough diagnosis. Additionally, only a small tissue fragment could be obtained and processed. To standardize identification and counting of myenteric neurons, the criteria published by Swaminathan et al., 2010 was applied [70]. In the hypertrophic and stenotic segments, twenty random fields of 160μm^2^ each were examined in the hematoxylin and eosin (H&E)-stained sections, as well as for those immunostained for calretinin, S-100 protein, CD56, neuron specific enolase (NSE) and synaptophysin for a total of 19.2mm^2^ of tissue studied. Each observation area was photographed using an Olympus BX51 light microscope and the images were analyzed using ImageJ software (Image-Pro Plus Version 3.1; Media Cybernetics, Inc.). The results revealed that in the stenotic segment the average number of cells was 20.21 ± 7.7 vs 40.12 ± 10.57 found in the hypertrophic segment, representing a 50% reduction in the density of ganglion cells. The independent-sample Student t test was used to compare differences between the two segments. The cumulative probability showed a significant difference (*P* = 0.021). The histological findings of stenosis indicate a relationship with HSCR, because the microscopic analysis showed a marked reduction of ganglion cells in the tissue portion removed.

It is currently recognized that this disorder takes on distinct forms, although clinicians and pathologists consulted in veterinary medicine know little about Hirschsprung’s disease. The pattern of staining for most of the markers (e.g., calretinin, S-100 protein and CD56) suggests a diagnosis of HSCR [71, 72]. Immunostaining was negative for most specific markers for HSCR. However, the ganglion cells detected may have been immature and/or non-functional. It can be assumed that ganglion cells were absent along the fragment of stenosis. Contrarily, these cells were present in the anastomosis at the level of the ileum, allowing for the normal functioning of the small intestine.

Fortunately, surgical resection for removal of the aganglionic bowel and reconstruction of the intestinal tract is now possible because of recent technological advances. Consequently, mortality due to a gastrointestinal obstruction can be avoided in animal models and humans. Among the acute complications of this disease, if untreated, is rapid exhaustion induced by the inability of the organism to nourish itself. The consequence could be toxicity and death.

In the field of veterinary medicine, there are few publications of clinical cases involving pets born with rare pathologies, which owes itself to the poor prognosis and the high cost of treatment. Hence, most pet owners choose to euthanize. Hirschsprung’s disease has complex pathogenesis that is not yet completely understood. Further basic and clinical research is necessary to elucidate the relationship between stenosis and HSCR and explore the similarity of the pathological mechanisms in animal models and humans.

## Abbreviations

BDNF: Brain-derived neurotrophic factor
CD56: Neural cell adhesion molecule
DAB: 3,3′- diaminobenzidine tetrahydrochloride hydrate
ECE-1: endothelin converting enzyme 1
EDN3: Endothelin 3
EDNRB: Endothelin receptor type B
ENS: Enteric nervous system
GDNF: Glial-cell-line-derived neurotrophic factor
H&E: Hematoxylin and eosin
HSCR: Hirschsprung’s disease
HSCR1: Hirschsprung disease type 1
HSCR2: Hirschsprung disease type 2
IHC: Immunohistochemistry
MHS1: Hematoxylin solution, Mayer’s
NGF: Nerve growth factor
NSE: Neuron specific enolase
NT-3: Neurotrophin-3
RET proto-oncogene: Encodes a receptor tyrosine kinase
RTKs: Receptor tyrosine kinases
S-100: Family of low-molecular-weight proteins
SOX10: Transcription factor SOX-10 is a protein encoded by the SOX10 gene
SSHD: Skip segment-Hirschsprung’s disease
SYN: Synaptophysin
